# Phase Error Reduction for a Structured-Light 3D System Based on a Texture-Modulated Reprojection Method

**DOI:** 10.3390/s24072075

**Published:** 2024-03-24

**Authors:** Chenbo Shi, Zheng Qin, Xiaowei Hu, Changsheng Zhu, Yuanzheng Mo, Zelong Li, Shaojia Yan, Yue Yu, Xiangteng Zang, Chun Zhang

**Affiliations:** 1 College of Intelligent Equipment, Shandong University of Science and Technology, Tai’an 271019, China; shichenbo@gmail.com (C.S.); qinz0304@163.com (Z.Q.); cs.zhu@sdust.edu.cn (C.Z.); 202283230023@sdust.edu.cn (Y.M.); 202383230007@sdust.edu.cn (Z.L.); 202383230010@sdust.edu.cn (S.Y.); 202383230013@sdust.edu.cn (Y.Y.); zangxt@sdust.edu.cn (X.Z.); 2 Department of Electronic Engineering, Tsinghua University, Beijing 100084, China; xiaoweihu1020@163.com

**Keywords:** phase-shifting method, phase error, texture edge, reprojecting, scene modulation

## Abstract

Fringe projection profilometry (FPP), with benefits such as high precision and a large depth of field, is a popular 3D optical measurement method widely used in precision reconstruction scenarios. However, the pixel brightness at reflective edges does not satisfy the conditions of the ideal pixel-wise phase-shifting model due to the influence of scene texture and system defocus, resulting in severe phase errors. To address this problem, we theoretically analyze the non-pixel-wise phase propagation model for texture edges and propose a reprojection strategy based on scene texture modulation. The strategy first obtains the reprojection weight mask by projecting typical FPP patterns and calculating the scene texture reflection ratio, then reprojects stripe patterns modulated by the weight mask to eliminate texture edge effects, and finally fuses coarse and refined phase maps to generate an accurate phase map. We validated the proposed method on various texture scenes, including a smooth plane, depth surface, and curved surface. Experimental results show that the root mean square error (RMSE) of the phase at the texture edge decreased by 53.32%, proving the effectiveness of the reprojection strategy in eliminating depth errors at texture edges.

## 1. Introduction

**Figure 1:**
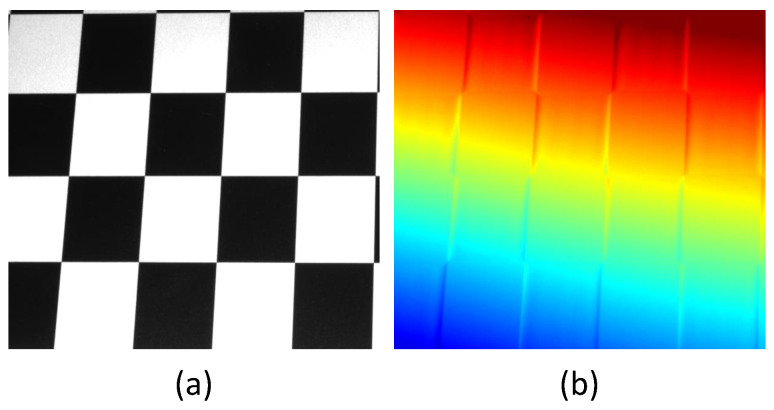
Measurement effect of traditional FPP method. (**a**) Smooth texture plane; (**b**) traditional FPP method measurement results.

As a non-contact 3D measurement technology, FPP [1] has been widely used in cultural relics protection, manufacturing, and other fields [2,3,4]. However, due to the influence of scene texture and imaging system defocus, the scene brightness does not meet the conditions of the ideal pixel-wise model at the edge of the reflectivity change, causing phase estimation errors, as shown in Figure 1. Figure 1a represents a smooth plane with texture, and Figure 1b is a depth image calculated using the traditional FPP method. As shown in Figure 1b, the brightness of the edge pixels is affected by the surrounding pixels due to the defocus of the image collected by the camera at the edge of the black-and-white texture, resulting in phase estimation errors. Therefore, improving the depth estimation accuracy at the texture edge is a difficult problem faced by the FPP method.

Defocus and the overall measurement accuracy of the system are the leading causes of discontinuity-induced measurement artifacts (DMAs). The generation of DMA errors can be slowed by improving the accuracy of the system measurement and the degree of defocus. To improve accuracy, Zhang et al. [5] regarded the projector as an inverse camera to achieve high-precision measurement. Li et al. [6] used a distortion model to create distortion stripe patterns for projection, thereby reducing the measurement error caused by distortion. Peter et al. [7] located the error pixels and directly deleted them. Pan et al. [8] proposed a method to optimize the phase error by using harmonics iteratively. Wu et al. [9] and Burke et al. [10] used mathematical models and post-processing algorithms to eliminate the impact of DMA errors. Although the above method can improve the overall measurement accuracy, it could be more effective in eliminating measurement errors caused by sudden changes in reflectivity.

In reducing the degree of defocus, the error is generally reduced by estimating the blur kernel. Li et al. [11] and Drouin et al. [12] reduced measurement errors by calculating the point spread function. Yue et al. [13] estimated the error pixel value based on the phase values around the error pixel. Wu et al. [14,15] estimated the phase error caused by the PSF through deconvolution. Brakhage et al. [16] used intensity gradients to measure the possible locations of artifacts and then used Gaussian curves to eliminate phase errors. Wang et al. [17] proposed a method to modify the projection intensity and exposure time at the pixel level by estimating the object’s surface reflectance and ambient light. Although the above methods have improved results for simple scenes, they cannot adapt to complex textures due to the difficulty in accurately estimating the blur kernel. These methods are not able to sufficiently distinguish between texture edges and actual depth edges.

In industrial measurement, FPP technology can quickly obtain the surface topography of objects. However, phase errors can occur during the measurement process because of the object’s surface texture interference. Therefore, this paper eliminates texture errors by modulating the intensity of the scene texture captured by the camera.

This paper theoretically analyzes the phase model and proposes a brightness equalization strategy based on scene modulation. First, the modulation intensity image of the scene is calculated using the actual scene image, and the modulation mask image is made using the coarse phase map. Then, the mask image is fused with the original stripe image to obtain the reprojected image. Finally, the coarse and reprojected phases are fused to obtain the refined phase map. The proposed method significantly improved depth error estimation at texture edges.

The remainder of this paper is organized as follows. Section 2 explains the FPP decoding model under non-pixel-wise ideal imaging. Section 3 describes the system framework of the feedback modulation projection method. Section 4 shows the experimental results. Section 5 discusses the experiments, and finally, Section 6 summarizes this paper.

## 2. Principle

This study used a monocular structured-light system to perform a 3D reconstruction of a scene. The system comprises a camera, a projector, a computer, and other equipment. Gray code and phase-shifting patterns are combined to perform the 3D reconstruction, not only retaining the advantages of the phase-shifting method but also alleviating the drawbacks, improving the 3D reconstruction accuracy of the scene.

Three-dimensional phase-shifting structured light generally uses M-bit Gray code patterns to obtain the phase period and N-step phase-shifting stripe patterns to obtain the wrapped phase. Subsequently, the wrapped phase is unwrapped to obtain a continuous phase. The N-step phase-shifting lighting model [18] projected by the projector can be expressed by Equation (1),

(1) $$\begin{array}{c} I_{n}\left(p\right)=A\left(p\right)+B\left(p\right)\cos\left[\varphi \left(p\right)+2n\pi /N\right] \end{array}$$

where *p* represents the pixel position, $A\left(p\right)$ represents the background light intensity, $B\left(p\right)$ represents the modulated light intensity, $\varphi \left(p\right)$ represents the wrapped phase of position *p*, *N* represents the number of phase-shifting steps, and *n* represents the *n*th phase-shifting fringe image.

**Figure 2:**
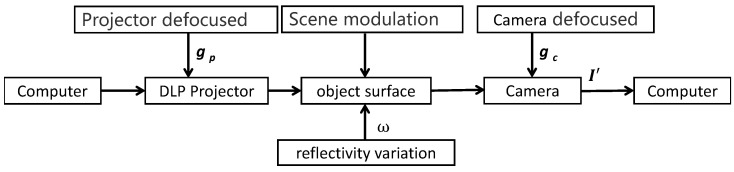
The process of capturing the intensity change in the stripe image by the camera.

However, during the measurement process, the local intensity of the target can change because of interference from factors such as camera defocus or sudden reflectivity changes, as shown in Figure 2.

According to Figure 2, when the camera is out of focus, the reflectivity of the measured surface changes. The light received by the camera will be affected, causing the intensity signal of a single pixel to be spatially averaged. Such pixels are called pixels affected by the non-pulse point spread function (PSF). The blur caused by defocus in the measurement system is similar to the Gaussian blur kernel [16]. Therefore, the defocus blur kernel $g_{c}$ can be expressed in the form of Equation (2),

(2) $$\begin{array}{c} g_{c}\left(x\right)=\frac{1}{\sqrt{2\pi }\sigma }e^{-\frac{\left(x^{2}\right)}{2\sigma^{2}}} \end{array}$$

where *x* represents the pixel position, and σ represents the Gaussian blur coefficient determined by the depth of the scene point. During the structured-light pattern’s projection, modulation, and collection process, the pattern experiences multiple interferences and conversions. Meanwhile, the image projected by the projector onto the target experiences interference, e.g., camera defocus and projector defocus. The image is also disturbed by sudden changes in the reflectivity of the object’s surface. Since the projector is out of focus, it does not directly affect the measured phase, but the sudden change in reflectivity will affect the measured scene. Assuming that point *q* is located in the neighborhood *D* of *p*, the actual brightness $I_{n}^{\prime }\left(p\right)$ of point *p* captured by the camera can be expressed in the form of Equation (3),

(3) $$\begin{array}{c} I_{n}^{\prime }\left(p\right)=\sum_{q\in D}I_{n}\left(q,p\right)\cdot g_{c}\left(q,p\right)=\sum_{q\in D}A\left(p\right)\cdot g_{c}\left(q,p\right)\\ +\sum_{q\in D}B\left(q\right)\cdot \omega \left(q\right)\cdot g_{c}\left(q,p\right)cos\left(\varphi \left(p\right)+\frac{2\pi n}{N}\right) \end{array}$$

where $B\left(q\right)$ represents the projection intensity of point *q*, w(q) represents the reflectivity of point *q*, and *n* represents the *n*th phase-shifting fringe pattern. The first term represents the influence of background light intensity; its value remains unchanged. Here, note $A^{\prime }\left(p\right)=\sum_{q\in D}A\left(p\right)\cdot g_{c}\left(q,p\right)$.

**Figure 3:**
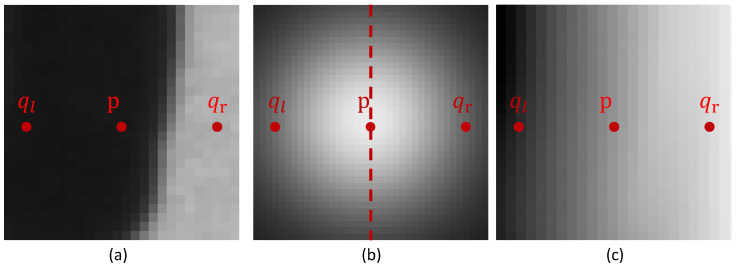
The relationship between camera defocus and phase in the scene. (**a**) The camera captures scene intensity; (**b**) the two-dimensional Gaussian distribution; (**c**) the phase value of (**a**).

For the second term in Equation (3), two points on the left and right sides of point *p* are picked in the scene in Figure 3a and are denoted by $q_{l}$ and $q_{r}$, respectively. Assuming that the formula in Equation (4) is established,

(4) $$\begin{array}{c} B\left(q_{l}\right)\cdot \omega \left(q_{l}\right)\approx B\left(q_{r}\right)\cdot \omega \left(q_{r}\right) \end{array}$$

and due to the symmetry properties of the Gaussian kernel in the local neighborhood, $g_{c}\left(q_{l},p\right)\approx g_{c}\left(q_{r},p\right)$ in Figure 3b. The relationship between the phases on point *p*’s left and right sides can be expressed in the form of Equation (5):

(5) $$\begin{array}{cc} \; & \cos\left(\varphi \left(q_{l}\right)+\frac{2\pi n}{N}\right)+\cos\left(\varphi \left(q_{r}\right)+\frac{2\pi n}{N}\right)=\\ & 2\cos\frac{\varphi \left(q_{r}\right)-\varphi \left(q_{l}\right)}{2}\cos\left(\frac{\varphi \left(q_{r}\right)+\varphi \left(q_{l}\right)}{2}+\frac{2\pi n}{N}\right) \end{array}$$

Because of the smoothness of the phase in Figure 3c, assuming $\varphi \left(q_{l}\right)+\varphi \left(q_{r}\right)\approx 2\varphi \left(p\right)$, we can obtain Equation (6):

(6) $$\begin{array}{c} \cos\left(\frac{\varphi \left(q_{r}\right)+\varphi \left(q_{l}\right)}{2}+\frac{2\pi n}{N}\right)\approx \cos\left(\varphi \left(p\right)+\frac{2\pi n}{N}\right) \end{array}$$

By combining Equations (3) and (6), the light intensity formula in the actual scene captured by the camera can be expressed as Equation (7):

(7) $$\begin{array}{c} I_{n}^{\prime }\left(p\right)=A^{\prime }\left(p\right)+\sum_{q\in \frac{D}{2}}B\left(q\right)\cdot \omega \left(q\right)\cdot g_{c}\left(q,p\right)\\ \cdot \cos\frac{\varphi \left(q_{r}\right)-\varphi \left(q_{l}\right)}{2}\cos\left(\varphi \left(p\right)+\frac{2\pi n}{N}\right) \end{array}$$

Since the value of $\varphi \left(q_{r}\right)-\varphi \left(q_{l}\right)$ is only related to depth changes in local scene points, it is irrelevant for $\varphi \left(p\right)$. Therefore, the factors independent of *n* in the second term of Equation (7) can be recorded in the form of Equation (8):

(8) $$\begin{array}{c} B^{\prime }\left(p\right)=\sum_{q^{\prime }\in \frac{D}{2}}B\left(q^{\prime }\right)\cdot \omega \left(q^{\prime }\right)\cdot g_{c}\left(q^{\prime },p\right)\cos\varphi \left(q^{\prime }\right) \end{array}$$

Hence, Equation (7) can be simplified to Equation (9):

(9) $$\begin{array}{c} I_{n}^{\prime }\left(p\right)=A^{\prime }\left(p\right)+B^{\prime }\left(p\right)\cos\left[\varphi \left(p\right)+2n\pi /N\right] \end{array}$$

A more accurate phase decoding result can be obtained by adopting the form of Equation (1).

(10) $$\begin{array}{c} \varphi \left(p\right)=arctan\frac{\sum_{n=0}^{n=N-1}I_{n}^{\prime }\left(p\right)\sin\frac{2\pi n}{N}}{\sum_{n=0}^{n=N-1}I_{n}^{\prime }\left(p\right)\cos\frac{2\pi n}{N}} \end{array}$$

The brightness model Equation (9) and the ideal phase model Equation (10) are based on the assumption of Equation (4). Therefore, to achieve brightness equalization for the scene, this paper proposes a feedback modulation projection strategy based on scene modulation to reduce the phase error at the texture edge.

## 3. Method

An anti-texture interference method based on feedback modulation projection is designed to correct measurement errors caused by a sudden change in reflectivity in the 3D reconstruction of structured light. Due to interference from the surface texture of the measured object, the pixels at the edge of the texture are disturbed by the pixels in the local neighborhood, which changes the ω value of the pixel and causes measurement errors. Inspired by this idea, this paper attempts to reduce the change degree of the ω value in a local neighborhood to reduce measurement errors.

### 3.1. Framework

**Figure 4:**
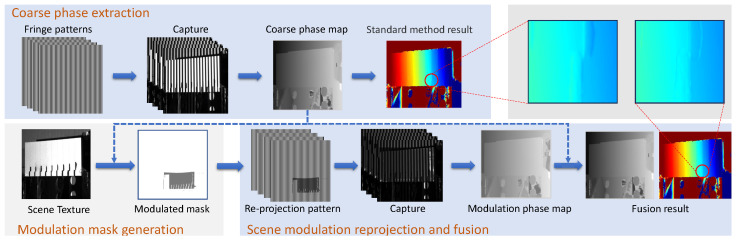
Computational framework of our proposed method.

The framework of the proposed method is shown in Figure 4. A typical method is used to obtain the original coarse phase value. The camera captures the most powerful illumination-modulated images to generate intensity-modulated images γ. According to the original coarse phase, the pixel positioning of the intensity-modulated image from the camera coordinate system to the projector coordinate system is realized, and the modulation mask image *M* is generated. *M* is combined with the original fringe to generate a reprojection pattern projected onto the scene to calculate the modulated absolute phase. The original coarse and modulated phases are fused to obtain the phase image with reduced error.

### 3.2. Modulation Mask Generation

To reduce the impact of reflectivity changes on structured-light 3D measurement, it is necessary to reduce the change in reflectivity at the edge of the texture. Hence, the illumination intensity of the scene captured by the camera becomes consistent and achieves uniform reflectivity, thus reducing measurement errors caused by sudden changes in reflectivity.

Regarding the distribution of error pixels in the original error image, this paper locates the error pixel position $I_{e}\left(p\right)$ through the edge intensity characteristics of the texture position in the maximum light modulation pattern. For the modulation strategy, the measurement system parameters, or light source intensity, are modified to reduce the projection intensity of pixels in high-reflectivity areas so that the reflectivity at the position of the sudden change tends to be uniform. That is, the intensities of the bright and dark areas captured by the camera are the same. Since this paper uses the maximum light intensity modulation pattern to calculate the modulation intensity, the modulation strategy is as shown in Equation (11):

(11) $$\begin{array}{c} \gamma \left(p\right)=\frac{cI_{k}^{\prime }\left(p\right)}{255\times \max\left(I_{l,i}\left(p\right),t\right)} \end{array}$$

where γ(p) represents the modulation intensity that the pixel needs to be projected, $I_{k}^{\prime }\left(p\right)$ represents the intensity value of point *p* in the pattern modulated by pure white light, and *t* represents a customized modulation intensity parameter. The purpose is to adjust the modulation intensity. $I_{l,i}\left(p\right)$ represents the lowest camera capture intensity, and *c* represents the self-set intensity modulation threshold. Such a modulation strategy can adjust the intensity of the projection parameters of the light source to meet the control requirements for sudden changes in reflectivity at the edge of the texture.

The projection modulation intensity information γ based on the camera pixel plane can be obtained by measuring changes in the scene surface reflectance. We also establish the mapping relationship between the camera and projector pixel coordinates. The texture edge correspondence during reprojection is achieved. According to the coarse phase, we can match the corresponding positions of the camera and the projector planes to obtain the modulated mask image *M*.

### 3.3. Scene Modulation Reprojection

**Figure 5:**
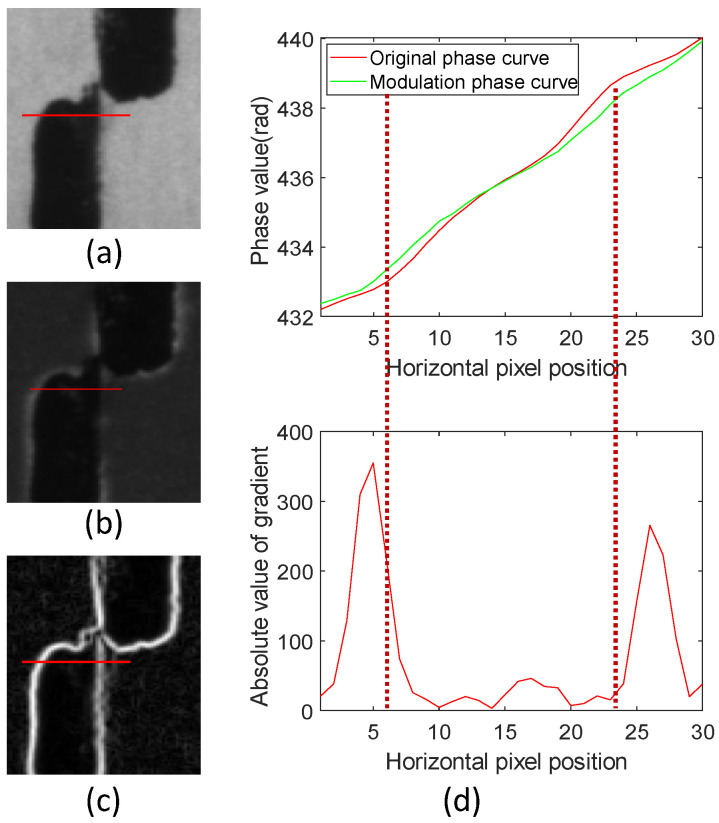
Modulation mask. (**a**) Maximum light modulation pattern; (**b**) scene image after adding mask; (**c**) gradient absolute value image; (**d**) absolute gradient value and phase comparison of line drawing positions.

By locating the error area and using the modulation strategy proposed in this paper, the ROI area scene shown in Figure 5a is intensity-modulated according to Equation (11). Image *M* is projected onto the surface of the measurement scene, as shown in Figure 5b. Before projecting image *M*, a rough estimate of the depth is required. When the depth estimate is inaccurate, it will cause a mismatch between the size of *M* and the texture of the scene. Image *M*’s size can be changed through morphological operations, such as expansion and erosion, during image processing.

The comparison in Figure 5d shows that image *M* can slow down the brightness change in the texture’s edge area. According to the comparison of the curves corresponding to the red line position in the image in Figure 5a–c, as shown in Figure 5d, the phase error peak appears on the darker side of the texture edge and gradient edge. Then, we used Equation (12) to add *M* to the original phase-shifting fringe pattern to obtain a new set of scene-modulated fringe patterns:

(12) $$\begin{array}{c} I_{r}\left(i\right)=I_{o}\left(i\right)\cdot M \end{array}$$

where $I_{o}\left(i\right)$ and $I_{r}\left(i\right)$, respectively, represent the *i*th original stripe pattern and the *i*th modulated stripe pattern in the projection sequence. After adding mask modulation, the change in the $\omega \left(p\right)$ value at the texture’s edge is reduced.

The generated stripe image with *M* is projected and collected. At this time, the reflectivity at the texture’s edge in the scene tends to be uniform. The camera obtains a scene image with uniform brightness when capturing the image. We decoded the reprojected image and calculated the new absolute phase value $\Phi_{r}$.

The phase error at the texture edge is reduced by fusing the original phase information with the modulated phase information. It can be seen from the comparison in Figure 5d that the error peak in the texture from the light to dark area appears on the dark side, with the peak value of the absolute gradient value as the divider; the error peak in the texture from the dark to light area is the same. Therefore, the average of absolute gradient values $G\left(p\right)$ in the local neighborhood is calculated using Equation (13) based on the pattern’s texture characteristics under the strongest illumination modulation and the gradient edge characteristics:

(13) $$\begin{array}{c} G\left(p\right)=\frac{\sum_{k=-m}^{m}\left|g\left(p+k\right)\right|}{2m+1} \end{array}$$

where g(p) represents the gradient value at position *p*, and *m* represents the neighborhood range for calculating the average of absolute gradient values.

We search for the depth peak in the original depth map following the position where the larger G(p) occurs in the dark area’s local neighborhood and find the position of the error pixel point $I_{e}\left(p\right)$. Phase fusion is performed according to the error pixel positioning. If a particular pixel is an error pixel, the modulated phase value is used as the fusion phase value. Otherwise, the original phase value is used, as shown in Equation (14):

(14) $$\begin{array}{c} \Phi_{f}\left(p\right)=\left\{\begin{array}{cc} \Phi_{o}\left(p\right) & \;\mathrm{if}\;\;I_{e}\left(p\right)=255,\\ \Phi_{r}\left(p\right) & \mathrm{if}\;\;I_{e}\left(p\right)=0. \end{array}\right. \end{array}$$

where $\Phi_{o}$ represents the original absolute phase map, and $\Phi_{f}$ represents the fused absolute phase map. Finally, the fused phase map is converted into height to obtain a depth map with reduced error.

**Figure 6:**
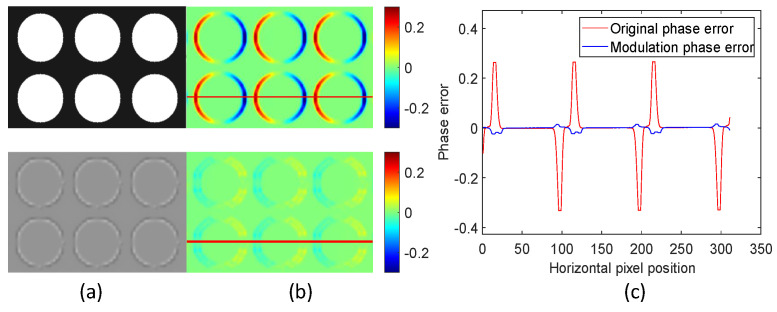
Phase error analysis of simulated modulated scene. (**a**) Simulation of original and modulated measurement scene pictures; (**b**) original phase error and modulated phase error; (**c**) comparison of the phase error.

To better analyze the phase error caused by texture, we utilized a fitting method to conduct phase error analysis. The phase error map is calculated by performing plane fitting on the acquired phase map using a nonlinear polynomial fitting method. Figure 6 depicts where the phase error occurs during reconstruction with the traditional structured-light method. Figure 6a compares the overall brightness of the original and modulated scenes. The brightness of the modulated scene is more uniform than that of the original scene.

Figure 6b shows the phase error map of the scene in Figure 6a, which is calculated using the traditional structured-light method and the proposed method. A conclusion can be drawn that the position where the phase error occurs in the scene is at the texture edge where the reflectivity changes. The modulated scene’s phase error is greatly reduced, as shown in the button of Figure 6b. Figure 6c compares the original phase error at the line drawing position in the scene with the modulated phase error. The phase error at the edge position is greatly reduced. The original phase error is 0.056 rad, and the phase error after modulation is 0.016 rad, decreasing by 71.43% compared to the original phase error. Although the error elimination effect of this method is relatively good, it will still produce considerable noise when the overall brightness of the scene is very dark.

## 4. Experiment

**Figure 7:**
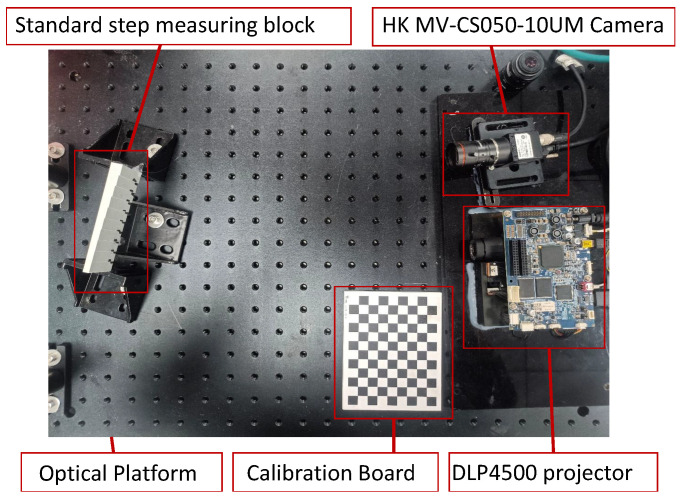
Structured-light 3D reconstruction system platform.

**Figure 8:**
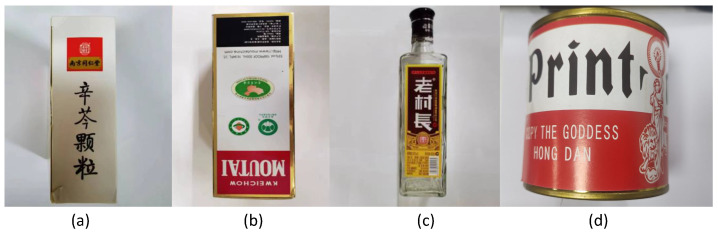
Actual measurement objects. (**a**) Smooth scene with only texture edges; (**b**) Scenes affected by both depth edges and texture edges; (**c**) Smooth surfaces affected by only texture edges; (**d**) Scenes with different depths of field.

This study used an industrial camera (resolution 2448×2048) and a optical machine (resolution 912×1140) to build a structured-light 3D reconstruction system, as shown in Figure 7. The phase-shifting fringe patterns and Gray code patterns in the horizontal and vertical directions were photographed, decoded, and reconstructed as the original control data. We then used the maximum light intensity modulation pattern to create a modulation projection mask pattern, added the modulation projection mask pattern to the phase-shifting stripe pattern, and added the modulation pattern to re-shoot the projection. The actual measurement scene is shown in Figure 8. Figure 8a,c are two scenes affected only by texture edges. Figure 8b shows a scene affected by both depth and texture edges. The white ‘MOUTAI’ words stand out, particularly in the background. Figure 8d depicts a cylindrical container used to verify the measurement effect under different depths of field.

### 4.1. Standard Step Measuring Block

**Figure 9:**
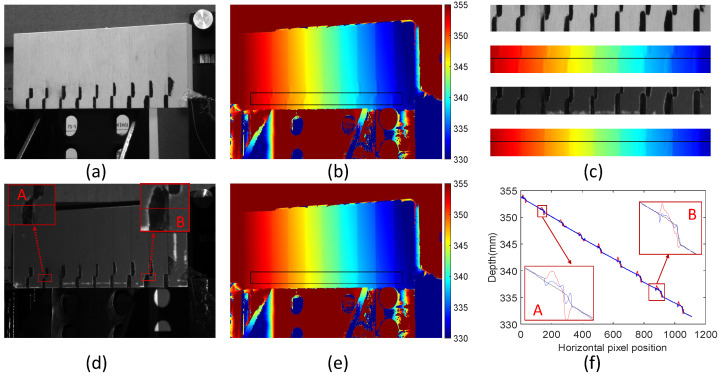
Comparison of measurement results for different depth differences. (**a**) Original scene image; (**b**) original depth map; (**c**) comparison of local ROI regions; (**d**) modulated scene image; (**e**) fusion depth map; (**f**) comparison of original depth curve (red), fusion depth curve (blue), and ground truth (black).

First, we tested the performance of the proposed scene modulation method on a standard step measuring block with texture. The height difference between every two steps of the standard step block increases by 0.1mm from left to right, and the height difference increases from 0.1mm to 0.9mm, as shown in Figure 9a.

We applied black paint at the edges of varying depths to create measured scenes affected by depth and texture. The depth map shown in Figure 9b was calculated using the traditional structured-light phase-shifting method. The proposed method performs feedback modulation on the measurement scene, as shown in Figure 9d. After scene modulation, the brightness difference among different reflectivities at the texture edge becomes smaller. The phase fusion method generates the fused depth map, as shown in Figure 9e. The depth error at the edge position of the black–white texture of the fused depth map is greatly reduced.

Figure 9c shows the local ROI comparison of the original scene, original depth map, modulated scene, and fused depth map from top to bottom. The brightness of the modulated scene changes slowly relative to the original scene at texture edge positions. Compared with the original depth map, the depth value of the fused depth map is improved at the edge positions where texture and depth are jointly affected. The depth is compared on a straight line from the exact position of the original and fused depth maps. The fused blue depth curve is closer to the actual black depth curve than the original red depth curve, as shown in Figure 9f. Comparing the magnified positions of A and B in Figure 9d and Figure 9f, it can be seen that the brightness change at the texture edge after modulation is less at position A than at position B. Therefore, the fusion effect at position A is much better.

The experiments show that the proposed method reduces the measurement error of edge positions affected by depth and texture in actual standard step measuring block scene modulation. Table 1 provides statistics on edge errors at different depths. The RMSE of the original measurement scene is 0.274 mm, and the RMSE after fusion is 0.114 mm, dropping by 58.39%.

### 4.2. Scene with Only Texture Edges

**Figure 10:**
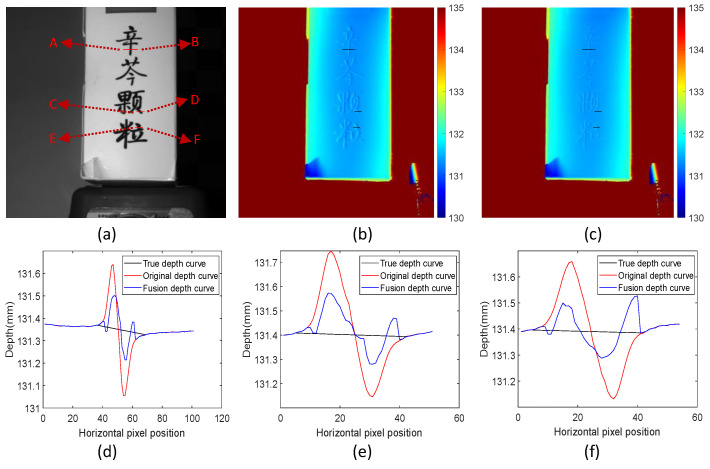
Comparison of measurement scenes only modulated by texture. (**a**) Original measurement scene; (**b**) original depth map; (**c**) fusion depth map; (**d**) depth comparison between position A and position B; (**e**) depth comparison between position C and position D; (**f**) depth comparison between position E and position F.

This study performed a structured-light 3D reconstruction of a measurement scene that is only affected by texture edges. The effectiveness of the proposed method is shown in Figure 10a. The scene has a black foreground and a white background. The proposed method was used for deep fusion, and the results are in Figure 10c. According to the comparison between the original depth map in Figure 10b and Figure 10c, a conclusion can be drawn that the method can significantly reduce the measurement error of texture edges.

The six original depth curves, fused depth curves, and actual reference values with different directional texture characteristics, as shown in Figure 10a, are compared in Figure 10d–f. Scenes A, C, and E represent black–white edges, and the remaining positions represent white–black edges. By comparison, it is found that the proposed method has similar effects when processing black–white textures and white–black textures and can reduce measurement errors at both edge positions. Since Figure 10d shows the texture change effect in the horizontal direction, and Figure 10e,f show the texture changes with different tilt degrees, the analysis of the curves shows that the proposed method is suitable for eliminating texture edge errors with different tilt degrees.

The data in Table 2 show that the proposed method is adaptable to both black–white and white–black types of edges, and the error compensation values for the two types are similar. The experimental results show that the proposed method can improve the accuracy of 3D measurements of scene surfaces modulated only by texture edges. According to the statistical analysis in Table 2, the original RMSE of this scene is 0.084 mm, and the fused RMSE is 0.039 mm. The error is reduced by 53.57%.

### 4.3. Scenes with Both Depth and Texture Edges

**Figure 11:**
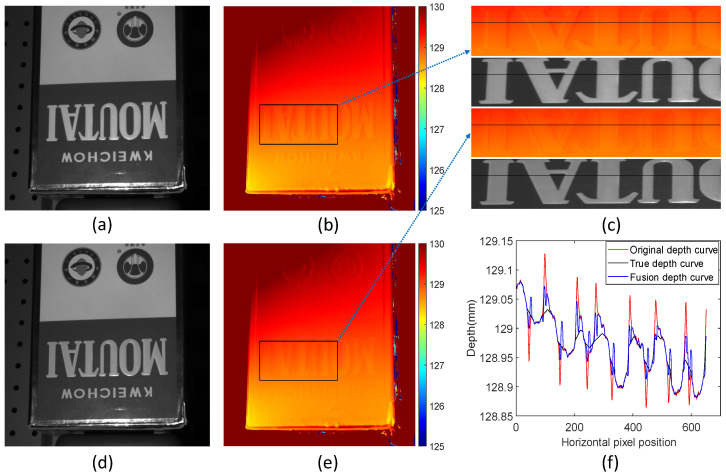
Comparison of measured effects on scenes modulated by depth and texture. (**a**) Original scene image; (**b**) original depth map; (**c**) comparison of local ROI regions; (**d**) modulated scene image; (**e**) fusion depth map; (**f**) comparison of original depth curve, modulated depth curve, and true depth curve.

This study performed a structured-light 3D reconstruction of a scene with both depth and texture edges, as shown in Figure 11a. The white ‘MOUTAI’ words stand out in this scene with a non-ideal step depth, particularly in the background. Figure 11b represents the original depth map. The original scene is intensity-modulated to obtain the modulated mask scene image, as shown in Figure 11d. In this image, the brightness change at the texture edge position is reduced relative to the change in the original scene.

Figure 11e depicts the results of the proposed fusion method. The depth error is significantly reduced at edge locations that are co-affected by depth and texture. The same ROI area is intercepted from the original scene image in Figure 11a, the modulated scene image in Figure 11d, the original depth map in Figure 11b, and the fused depth map in Figure 11e for comparison, as shown in Figure 11c. It can be seen that the brightness of ‘MOUTAI’ in the modulated scene became dark and that the scene’s contrast was reduced, and we can see the change in the depth value.

This paper analyzes the depth information of the original depth map and the fused depth map at the position of the black line. As shown in Figure 11f, it can be intuitively seen that the error of the fused depth curve is significantly reduced compared to the original curve. The original RMSE of the scene jointly affected by depth and texture is 0.011 mm, and the fused RMSE is 0.006 mm, with the error reduced by 45.45%. The experiments show that the fused depth map can significantly reduce the measurement error of edges jointly affected by depth and texture.

### 4.4. Scenes with Textures of Different Widths

**Figure 12:**
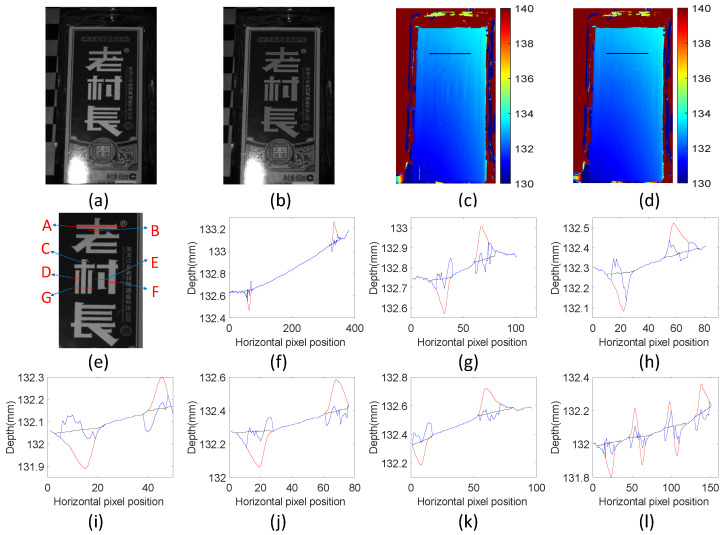
Comparison of measurements of different texture widths. (**a**) Original measurement scene; (**b**) modulated scene image; (**c**) original depth map; (**d**) fusion depth map; (**e**) ROI of original measurement scene; (**f**–**l**) original depth (red), fusion depth (blue), and actual value (black) comparison at positions A–G in (**e**).

This study performed a structured-light 3D reconstruction of a scene with texture characteristics of different widths, as shown in Figure 12a. The scene is a measurement surface with a white foreground and a dark background, only affected by texture edges. After scene modulation in Figure 12b, the pixel intensities at texture edge positions with different widths in the scene are well modulated. The traditional method and the proposed method were each used for the depth calculation, as shown in Figure 12c,d. The comparison shows that the proposed method is also suitable for the scene measurement of different widths.

The original depth curves, fused depth curves, and corresponding actual reference values of different line drawing positions in the ROI area were analyzed, and the ROI image is shown in Figure 12e. Positions (f)–(l) in Figure 12 are the positions represented by A–G in Figure 12e. The texture widths at these positions are different. Among them, positions A–F contain only one texture in the background. There is no interference from other textures near the texture, so these positions are less affected by textures at other positions. Therefore, the fusion effect at these locations is relatively ideal. The edge of the texture at position G is compact and is significantly interfered with by surrounding pixels compared to positions A–F.

Table 3 compares the RMSE values of the seven positions (f)–(l) in Figure 12. The original RMSE of the measurement scene is 0.068 mm, and the fused RMSE is 0.030 mm, decreasing by 55.88% on average. The experiments show that the proposed method is effective in the surface measurement of objects with texture edges of different widths.

### 4.5. Analysis of Different Depths of Field

**Figure 13:**
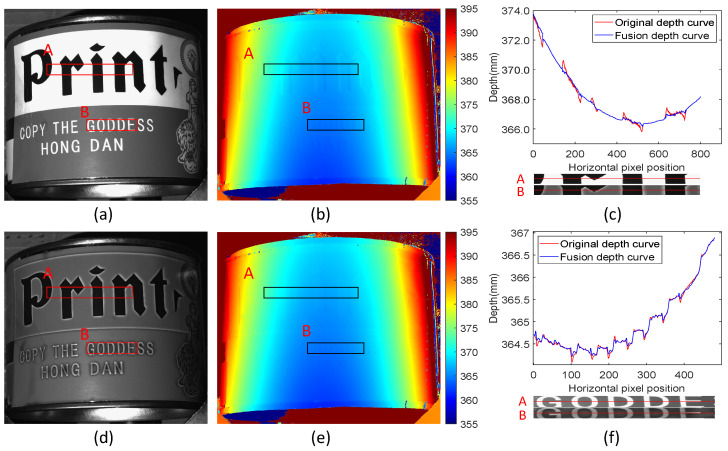
Measurement experiments under different depths of field. (**a**) The original scene image; (**b**) the original depth map; (**c**) the original depth map compared with the depth curve of position A in the fusion depth map; (**d**) the modulated scene image; (**e**) the fusion depth map; (**f**) the original depth map compared with the depth curve of position B in the fused depth map.

This study used the scene shown in Figure 13a for an experimental comparison, and the traditional method was applied to calculate the depth map shown in Figure 13b. Since the object’s surface is cylindrical, different camera defocus blur kernels will affect different positions during the measurement process. The proposed method was used to perform feedback modulation projection of the scene, as shown in Figure 13d. After scene modulation, the intensity of the white portion captured by the camera became darker. The depth map shown in Figure 13e was calculated using the proposed method in this paper.

Figure 13c compares the depth curves at position A in the original and fused scenes. In Figure 13c, A represents the texture ROI area in the original scene, and B represents the ROI area in the modulated scene. Since the camera on the left side of the scene has the most accurate focus, the left side is less affected by the camera’s out-of-focus PSF, thus making the fusion effect more accurate. Figure 13f compares the depth curves of position B in the original and fused depth maps. Since position B is closer to the camera and projector than position A in the scene, the light intensity received by position B is stronger. The modulation effect at position B captured by the camera is not relative to the original scene A, so the fusion effect at position A is better than that at position B.

The experimental results show that the proposed method is suitable for scene measurement with different depths of field. However, the greater the camera defocus, the stronger the interference from the scene PSF. Therefore, the more accurately the camera focuses on the scene, the more pronounced the error elimination effect.

## 5. Discussion

**Figure 14:**
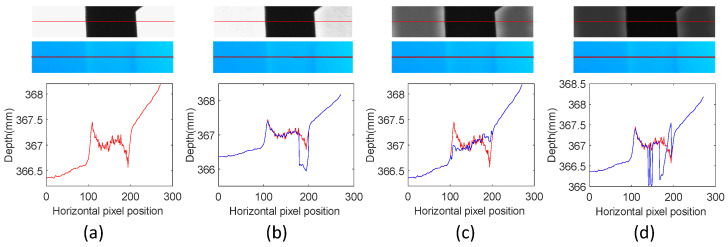
Comparison of the modulation effects of different light intensities. (**a**) The reconstruction effect of the traditional method; (**b**–**d**) the reconstruction result when the modulated light intensity is 220, 90, and 50.

This paper proposes a feedback modulation projection strategy based on scene intensity modulation to address the error problem at texture edges. Projectors of different intensities produce different effects. Figure 14 compares the scene effects after different intensity modulations. Figure 14a shows the measurement results obtained with the standard measurement method. From top to bottom, the figure shows the camera-captured light intensity, the depth map under the current intensity, and the depth curve of the line drawing positions. When the projector projects a light intensity of 220, as shown in Figure 14b, the contrast between light and dark edges does not change significantly. Thus, the measurement effect is poor when the projected light intensity is 90, as shown in Figure 14c. Depth errors at texture edges are greatly reduced when the projection intensity is 50, as shown in Figure 14d. There is considerable noise in the calculated depth map due to the dimming of the light intensity in the entire scene. Therefore, the light projected by the projector must meet the condition that the intensity on both sides of the texture edge captured by the camera be even to achieve the best fusion effect.

Although the method proposed in this article can effectively reduce the phase errors at texture edge positions, the method still needs improvement in many aspects. First of all, the measurement method proposed in this article has many system parameters, and some parameters need to be adjusted manually, making the parameter adjustment process cumbersome. Secondly, during the fusion process, the fusion method proposed in this article still has inaccurate positioning for the error pixel position. Finally, due to differences in camera projector resolution, there are errors in texture edge positioning when making reprojected patterns.

## 6. Conclusions

This paper proposes a 3D reconstruction method to mitigate texture interference based on feedback modulation projection. This method eliminates phase errors caused by defocus and sudden changes in reflectivity at the edge of texture. According to the change in the reflectivity coefficient in the measurement scene, consistent reflectivity is achieved by changing the projection intensity and eliminating the measurement error caused by a sudden change in the reflectivity of the texture edge. The experimental results show that the error of the edge position is reduced by optimizing the feedback modulation projection strategy on the measured surface, which is only affected by the texture edge. Plenty of experimental data prove that the root mean square error of the proposed method can be reduced by 53.32% on average.

## Figures and Tables

**Table 1:** RMSE comparison of standard ladder blocks with different depth differences.

Depth Difference/(mm)	0.1	0.2	0.3	0.4	0.5	0.6	0.7	0.8	0.9
Original RMSE (mm)	0.256	0.279	0.189	0.206	0.307	0.329	0.319	0.294	0.287
Fusion RMSE (mm)	0.116	0.114	0.104	0.124	0.102	0.099	0.111	0.117	0.141
Improvement	54.69%	59.14%	44.97%	39.81%	66.78%	69.91%	65.20%	60.20%	50.87%

**Table 2:** RMSE comparison of scenes modulated only by texture.

Number	A	B	C	D	E	F
Original RMSE (mm)	0.058	0.063	0.091	0.086	0.120	0.086
Fusion RMSE (mm)	0.032	0.025	0.034	0.047	0.056	0.038
Improvement	44.83%	60.32%	62.64%	45.35%	53.33%	55.81%

**Table 3:** RMSE comparison of scenes with different texture characteristics.

Number	A	B	C	D	E	F	G
Original RMSE (mm)	0.037	0.062	0.070	0.073	0.088	0.065	0.081
Fusion RMSE (mm)	0.015	0.027	0.034	0.035	0.033	0.032	0.036
Improvement	59.46%	56.45%	51.43 %	52.05%	62.50%	50.77%	55.56%

## Data Availability

Data are contained within the article.
